# Anti-Biofouling Electrochemical Sensor Based on the Binary Nanocomposite of Silica Nanochannel Array and Graphene for Doxorubicin Detection in Human Serum and Urine Samples

**DOI:** 10.3390/molecules27248640

**Published:** 2022-12-07

**Authors:** Ning Lv, Xun Qiu, Qianqian Han, Fengna Xi, Yina Wang, Jun Chen

**Affiliations:** 1 Department of Pharmacy, The First Affiliated Hospital, College of Medicine, Zhejiang University, 79 Qingchun Road, Hangzhou 310003, China; 2 Key Laboratory of Integrated Oncology and Intelligent Medicine of Zhejiang Province, Department of Hepatobiliary and Pancreatic Surgery, Affiliated Hangzhou First People’s Hospital, Zhejiang University School of Medicine, Hangzhou 310006, China; 3 Department of Chemistry, Zhejiang Sci-Tech University, Hangzhou 310018, China; 4 Department of Medical Oncology, The First Affiliated Hospital, College of Medicine, Zhejiang University, 79 Qingchun Road, Hangzhou 310003, China

**Keywords:** silica nanochannel array, electrochemically reduced graphene oxide, screen-printed carbon electrode, doxorubicin, electrochemical sensor

## Abstract

A disposable and portable electrochemical sensor was fabricated by integrating vertically-ordered silica mesoporous films (VMSF) and electrochemically reduced graphene (ErGO) on a screen-printed carbon electrode (SPCE). Such VMSF/ErGO/SPCEs could be prepared by a simple and controllable electrochemical method. Stable growth of VMSF on SPCE could be accomplished by the introduction of an adhesive ErGO nanolayer owing to its oxygen-containing groups and two-dimensional (2D) planar structure. An outer VMSF layer acting as a protective coating is able to prevent the leakage of the inner ErGO layer from the SPCE surface. Thanks to the electrostatic permselectivity and anti-fouling capacity of VMSF and to the good electroactive activity of ErGO, binary nanocomposites of VMSF and ErGO endow the SPCE with excellent analytical performance, which could be used to quantitatively detect doxorubicin (DOX) in biological samples (human serum and urine) with high sensitivity, good long-term stability, and low sample amounts.

## 1. Introduction

Doxorubicin (DOX) is a kind of broad-spectrum anticancer drug that has been widely used in clinical treatment of various cancers, such as breast and lung cancer, Hodgkin’s and non-Hodgkin’s lymphomas, and sarcomas. However, long-term use of DOX or excess DOX in the human body will cause gastrointestinal wounds, liver failure, severe cardiotoxicity, nephrotoxicity [1], myelosuppression, or cardiomyopathy [2]. Therefore, analytical determination of DOX in biological samples is of great importance. At present, approaches for DOX detection mainly contain capillary electrophoresis [3], fluorescence [4], solid-phase microextraction [5], surface-enhanced Raman spectroscopy [6], and electrochemical methods [7,8]. Among them, electrochemical sensors are advantageous in terms of their low cost, high sensitivity, and easy operations, which is compatible with portable point-of-care testing and on-the-spot analysis. [9,10,11] However, electrochemical detection is often limited to simple samples and suffers from the electrode biofouling in complicated biological fluids (e.g., whole blood and urine). Direct electrochemical analysis of complicated biological samples is usually performed by combination with an anti-fouling protective coating, such as porous membranes [12], polyethylene glycol [13], nanoporous gold [14], and others [15,16].

Vertically-ordered mesoporous silica films (VMSF), or so-called silica nanochannel arrays, have received great attention in past decades for their perpendicular nanochannels, molecular permselectivity and anti-fouling capacity in direct analysis of complicated real samples without further tedious pretreatments [17]. The excellent anti-fouling capacity of VMSF arises from its ultrasmall and uniform pore size, tailored lipophilicity/charge selectivity, and insolated properties. Various targeted analytes, ranging from ions [18,19], small biological molecules [20,21], explosives [22], environmental pollutants [23,24,25,26], drugs [27,28,29,30], and biomarkers [31,32,33,34,35], can be detected by VMSF-based sensors. Electrochemically-assisted self-assembly (EASA) approach is considered as the most rapid method for the growth of highly ordered VMSF on solid electrode substrates, such as glassy carbon electrodes, indium tin oxide, gold, platinum, screen-printed carbon electrodes, (SPCE) and other conductive electrodes [36,37,38,39]. Among them, SPCE exhibits great advantages in terms of its disposable and convenient use, miniaturization, and inexpensiveness, which is very suitable for utilization in the electrochemical analysis of real samples.

Graphene based nanomaterials have attracted increasing interest due to their unique structure, multidimensional scale manipulation (e.g., 0D quantum dots [40], 2D sheets [41], and 3D porous foam [42,43]), exceptional physicochemical properties and novel applications in energy storage and conversion, electro/photo/chemical catalysis, chemo-/bio-sensors [44,45,46] etc. In this work, we prepared a disposable and portable electrochemical sensor by integrating VMSF and electrochemically reduced graphene oxide (ErGO) on a SPCE. VMSF/ErGO/SPCE can be prepared by a controllable one-step electrochemical method, and it is able to quantitatively detect DOX by differential pulse voltammetry (DPV), which combines the electrostatic preconcentration capacity of VMSF and excellent electroactive activity of ErGO. In addition, thanks to the anti-fouling and anti-interference ability of VMSF, the developed VMSF/ErGO/SPCE can be applied for direct analysis of DOX in human serum and urine samples without complex sample pretreatments.

## 2. Results and Discussion

### 2.1. Fabrication and Characterizations of VMSF/ErGO/SPCE

**Figure 1:**
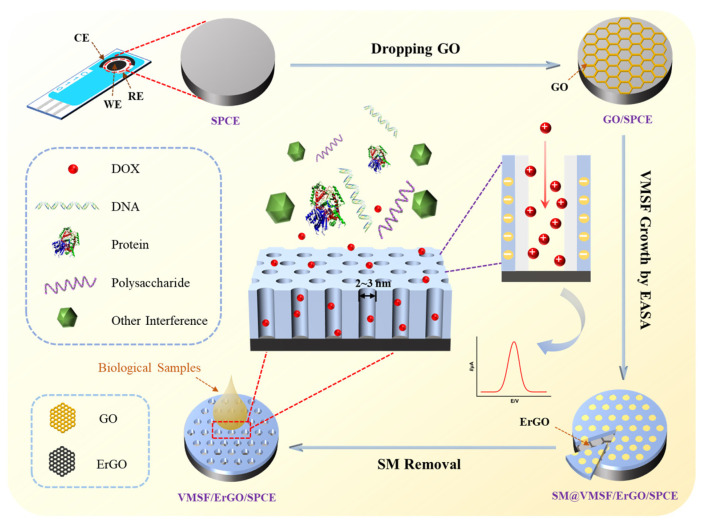
Schematic illustration for the preparation of VMSF/ErGO/SPCE and the direct detection of DOX in complex biological samples.

**Figure 2:**
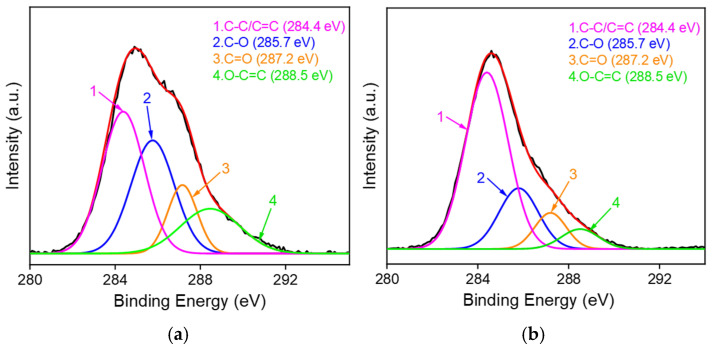
Carbon 1s XPS profiles of (**a**) GO/SPCE and (**b**) ErGO/SPCE.

As shown in Figure 1, GO on the SPCE surface could be electrochemically reduced to ErGO in the process of VMSF growth and can be characterized by X-ray photoelectron spectroscopy (XPS). As identified in the carbon 1s XPS profiles (Figure 2), GO/SPCE is characterized by four peak signals at 284.4 eV, 285.7 eV, 287.2 eV, and 288.5 eV, respectively, corresponding to the C-C/C=C, and three kinds of oxygen-containing groups (C-O, C=O, and O-C=O). Upon the electrochemical reduction of GO to ErGO, the intensities of oxygen-containing groups apparently decreases, suggesting the successful reduction of GO on the SPCE surface in the growth process of VMSF.

**Figure 3:**
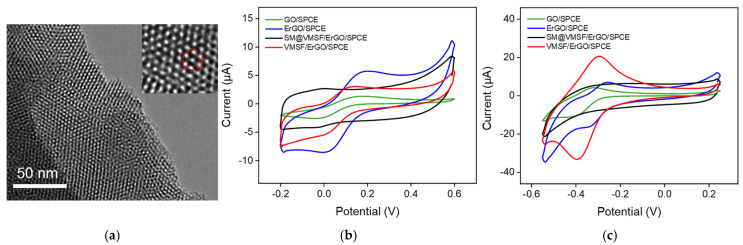
(**a**) Top−view TEM images of VMSF at different magnifications; CV curves of different electrodes (GO/SPCE, ErGO/SPCE, SM@VMSF/ErGO/SPCE, VMSF/ErGO/SPCE) in 50 mM potassium phthalate (KHP) solution containing 0.5 mM (**b**) K_3_[Fe(CN)_6_] and (**c**) Ru(NH_3_)_6_Cl_3_ at a scan rate of 50 mV/s.

VMSF grown on the ErGO/SPCE surface was confirmed by TEM and cyclic voltammetry (CV). The TEM image shown in Figure 3a indicates the VMSF has a high density of hexagonally packed silica nanopores and that the nanopores are very uniform with a diameter of 2~3 nm. Two charged electrochemical probes were used to examine the intactness and charge permselectivity of VMSF. Figure 3b,c show CV curves of Fe(CN)_6_^3−^ (b) and Ru(NH_3_)_6_^3+^ (c) for GO/SPCE, ErGO/SPCE, SM@ VMSF/ErGO/SPCE and VMSF/ErGO/SPCE. As seen, electrochemical reduction of GO to ErGO could greatly enhance the electrochemical signals of Fe(CN)_6_^3−^ and Ru(NH_3_)_6_^3+^, which is attributed to the good electrochemical activity of ErGO. The presence of SM inside the silica nanochannels results in the charging currents for both Fe(CN)_6_^3−^ and Ru(NH_3_)_6_^3+^, indicating the good integrity of the as-prepared VMSF on the ErGO/SPCE. After extraction of SM from the silica nanochannels, VMSF with open channels on the ErGO/SPCE favors the mass transport to the underlying electrode surface through the vertical nanochannels. In comparison with the ErGO/SPCE, VMSF/ErGO/SPCE displays improved redox current signals for positively charged Ru(NH_3_)_6_^3+^ and decreased signals for negatively charged Fe(CN)_6_^3−^, which is due to the deprotonation of silanol groups in VMSF in the experimental conditions. This charge permselectivity phenomenon of VMSF on the ErGO/SPCE is similar to those reported previously [11] and shows great potential for amplified current signals of cationic species.

### 2.2. Electrochemical Behavior of DOX at the VMSF/ErGO/SPCE

**Figure 4:**
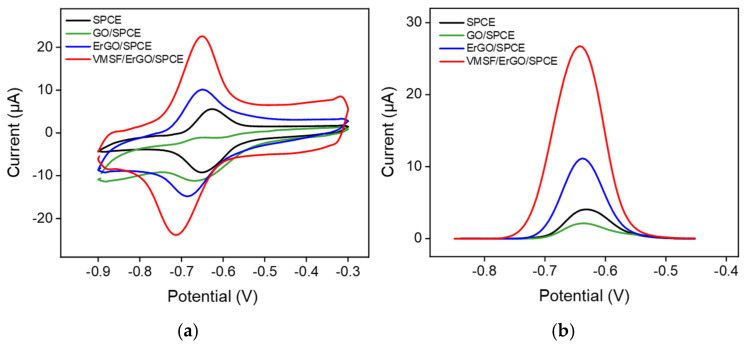
(**a**) CV and (**b**) DPV curves of bare SPCE (black), GO/SPCE (green), ErGO/SPCE (blue) and VMSF/ErGO/SPCE (red) in 0.1 M PBS (pH = 6.0) solution containing 10 μM DOX. The scan rate in (**a**) was 100 mV/s, and the potential in (**b**) ranged from −0.85 V to −0.45 V.

The electrochemical behavior of DOX for bare SPCE, ErGO/SPCE and VMSF/ErGO/SPCE was studied by CV and differential pulse voltammetry (DPV). As shown in Figure 4a, a pair of redox peaks is observed for bare SPCE, which is ascribed to the electron transfer between the quinone and hydroxyquinone groups of DOX. Due to the poor conductivity of GO, GO/SPCE shows a decreased redox current compared with the bare SPCE. Modification of ErGO on the SPCE results in the enhanced redox current signals, proving the good electroactivity of ErGO. After further growth of VMSF on the ErGO/SPCE, remarkably increased redox current signals are observed, which is due to the electrostatic enrichment of VMSF with negatively charged silica walls. It could be found that the magnitude of the current signal corresponding to the electrochemical oxidation of DOX for VMSF/ErGO/SPCE is 2.5-fold larger than that for ErGO/SPCE and 6.9-fold larger than that at the bare SPCE (Figure 4b), revealing that the integration of VMSF and ErGO could effectively improve the analytical performance of sensors towards DOX. Moreover, the effect of the scan rate on the CV curves of DOX is investigated in Figure S1, suggesting the electrochemical process of DOX on the VMSF/ErGO/SPCE is adsorption-controlled.

### 2.3. Analytical Performance of VMSF/ErGO/SPCE towards DOX

**Figure 5:**
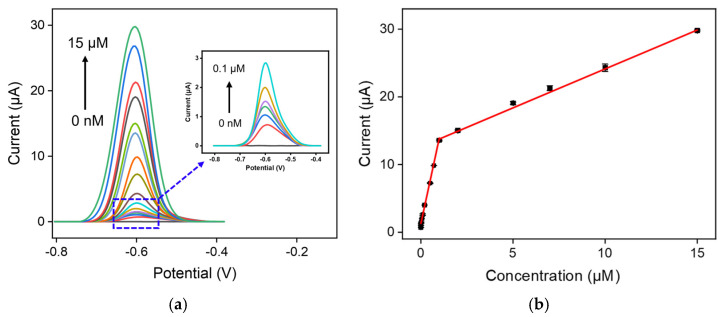
(**a**) DPV curves and (**b**) the calibration curve of VMSF/ErGO/SPCE in 0.1 M PBS solution (pH 6.0) containing DOX ranging from 0 nM to 15 μM (0, 0.002, 0.005, 0.01, 0.02, 0.05, 0.1, 0.2, 0.5, 0.7, 1, 2, 5, 7, 10, and 15 μM). Inset in (**a**) is the amplified view of the DPV curves in the low-concentration region and the potential ranged from −0.8 V to −0.4 V. The error bars in (**b**) represent the standard deviations of three measurements.

Prior to DOX detection, the influence of growth time of VMSF, preconcentration time, and pH of the supporting electrolyte were first examined: 10 s, 3 min, and pH = 6.0 are the optimal growth time of VMSF, preconcentration time, and pH of the supporting electrolyte, respectively (Figures S2 and S3). DPV responses of VMSF/ErGO/SPCE to various concentrations of DOX were recorded in Figure 5a. As shown, the anodic peak current of DOX increased obviously with increasing DOX concentration, yielding linear fitting curves over two dynamic ranges of 2 nM–1 μM and 1–15 μM (Figure 5b). The analytical sensitivities in the low and high concentration ranges are 12.6 μA/μM and 1.15 μA/μM, respectively. The appearance of two linear ranges is associated with the electrostatic interaction between cationic DOX and VMSF with negatively charged walls, resulting in the enrichment of DOX onto the electrode surface. The different enrichment abilities in the low and high concentration ranges leads to the different detection sensitivities (namely a larger slope in the low concentration range). The limit of detection (LOD) is calculated to be 1 nM. Analytical performance obtained for VMSF/ErGO/SPCE, including linear range, sensitivity, and LOD, are compared with those of other electrochemical sensors reported previously, as shown in Table 1. It could be found that our developed VMSF/ErGO/SPCE sensor exhibits improved sensitivity, wider linear range, and lower LOD. Note that our proposed VMSF/ErGO/SPCE has the advantages of disposable and convenient use, miniaturization, and inexpensiveness, which makes it more suitable for utilization in the electrochemical analysis of real samples, in comparison with the VMSF/ErGO/GCE.

### 2.4. Anti-Interference, Reproducibility and Stability of VMSF/ErGO/SPCE

**Figure 6:**
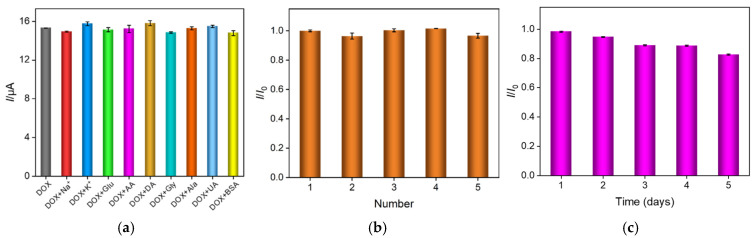
(**a**) The current ratio (*I*/*I*_0_) obtained from VMSF/ErGO/SPCE for the detection of 10 μM DOX in the absence (*I*_0_) and presence (*I*) of added interfering species. The concentration of BSA is 0.4 mg/mL and the concentration of other interfering species are 1 mM; (**b**) The current ratio (*I*/*I*_0_) obtained from five parallel VMSF/ErGO/SPCE for detection of 10 μM DOX—*I*_0_ and *I* represent the signals of the first electrode and other electrodes, respectively; (**c**) The stability of VMSF/ErGO/SPCE in the detection of 10 μM DOX for 5 days—*I*_0_ and *I* mean the current measured on the first day and subsequent days.

Some potential interferents, such as Na^+^, K^+^, glucose (Glu), ascorbic acid (AA), dopamine (DA), glycine (Gly), alanine (Ala), uric acid (UA), and bovine serum albumin (BSA), were considered as the coexisting species in the biological fluids and were used to evaluate the anti-interference ability of the VMSF/ErGO/SPCE on the DOX detection. As shown in Figure 6a, when 1 mM Na^+^, K^+^, Glu, AA, DA, Gly, Ala, UA, and 0.4 mg/mL BSA are present, the anodic peak current of DOX remains nearly unchanged, proving the good anti-interference capacity of the VMSF/ErGO/SPCE. In addition, reproducibility and stability of VMSF/ErGO/SPCE are demonstrated in Figure 6b,c. As seen, the obtained relative standard deviation (RSD) between five prepared VMSF/ErGO/SPCEs is 2.3% and the current associated with the oxidation of DOX remains 82.7% of its initial measured value after 5-day storage.

### 2.5. Real Sample Analysis

**Figure 7:**
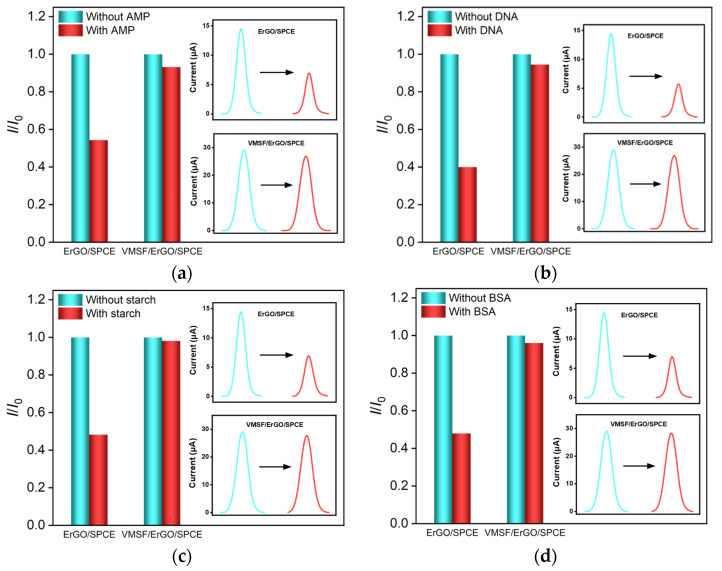
The peak current ratio (*I*/*I*_0_) of DOX (10 µM) for the ErGO/SPCE or VMSF/ErGO/SPCE in PBS (0.1 M, pH = 6) with (*I*) or without (*I*_0_) 10 μg/mL (**a**) AMP, (**b**) DNA, (**c**) starch, or (**d**) BSA. The insets are the corresponding DPV curves obtained for the ErGO/SPCE and VMSF/ErGO/SPCE in the absence (green curve) or presence (red curve) of fouling species.

Antimicrobial peptide (AMP), DNA, starch, and bovine serum albumin (BSA) were employed to confirm the anti-fouling property of VMSF/ErGO/SPCE by comparing the current signals obtained at the ErGO/SPCE, and the results are shown in Figure 7. As displayed in the insets in Figure 7, in the absence of the above fouling substances, both ErGO/SPCE and VMSF/ErGO/SPCE could give rise to the apparent anodic peaks for DOX and the respective signal for VMSF/ErGO/SPCE was much higher, which matches the results shown in Figure 4b. It was obvious that the presence of 10 μg/mL of these four biological macromolecules led to the decreased anodic peak currents at the ErGO/SPCE, however, no apparent variation was observed for the VMSF/ErGO/SPCE, indicating the excellent anti-fouling ability of the developed VMSF/ErGO/SPCE sensor. Moreover, determination of DOX in human serum and urine samples was demonstrated. Human serum and urine samples diluted by 50 times with 0.1 M PBS (pH = 6.0) without any further complex pretreatment were spiked with a known concentration of DOX and detected by the VMSF/ErGO/SPCE sensor. As displayed in Table 2, the obtained recoveries are from 99.2 to 104% and the value of RSD is less than 3.1%, showing that the proposed VMSF/ErGO/SPCE is capable of quantitative analysis of DOX in human serum and urine samples without complex sample pretreatment.

## 3. Materials and Methods

### 3.1. Chemicals and Materials

All chemicals and reagents were of analytical grade and did not undergo further treatment. All aqueous solutions were prepared using ultrapure water (18.2 MΩ cm). Doxorubicin (DOX), cetyltrimethylammonium bromide (CTAB), tetraethoxysilane (TEOS), potassium ferricyanide (K_3_[Fe(CN)_6_]), potassium ferrocyanide (K_4_[Fe(CN)_6_]), sodium phosphate monobasic dihydrate (NaH_2_PO_4_·2H_2_O), sodium phosphate dibasic dodecahydrate (Na_2_HPO_4_·12H_2_O), glucose (Glu), ascorbic acid (AA), uric acid (UA), dopamine (DA), glycine (Gly), and alanine (Ala) were purchased from Shanghai Aladdin Biochemical Technology Co., Ltd. (Hangzhou, China) Antimicrobial peptide (AMP) and starch were bought from Macklin (Shanghai, China). Deoxyribonucleic Acid (DNA) was obtained from Shanghai Sangon Biotech (Shanghai, China). Hexaammineruthenium (III) chloride (Ru(NH_3_)_6_Cl_3_) and bovine serum albumin (BSA) were received from Sigma Aldrich (Shanghai, China). Sodium nitrate (NaNO_3_), sodium chloride (NaCl), potassium chloride (KCl), and sodium hydroxide (NaOH) were purchased from the Hangzhou Gaojing Fine Chemical Reagent (Hangzhou, China). Ethanol and hydrochloric acid were obtained from Hangzhou Shuanglin Chemical Reagent Co., Ltd. (Hangzhou, China). NaH_2_PO_4_·2H_2_O and Na_2_HPO_4_·12H_2_O were mixed together in a definite proportion to prepare phosphate buffer solution (PBS).

Screen-printed carbon electrode (SPCE, DRP-C110-U75) with a three-electrode system was purchased from Metrohm (Bern, Switzerland), where carbon electrode was used as the working (4 mm in diameter) and counter electrodes, and silver as the reference electrode.

### 3.2. Instruments and Equipment

Transmission electron microscope (TEM) images were obtained at an acceleration voltage of 200 kV on a JEM-2100 transmission electron microscope (JEOL, Ltd., Tokyo, Japan). TEM sample preparation process was as follows: VMSF was gently scraped from the surface of SPCE into ethanol solution with a knife several times, and then the solution was uniformly dispersed by ultrasonic treatment; the obtained dispersed solution was dropped onto the copper net and dried, prior to the observation under a microscope. X-ray photoelectron spectroscopy (XPS; MA, USA) was performed on a PHI5300 electron spectrometer to analyze the functional groups of electrode materials, which was excited by a Mg Kα source at 250 W and 14 kV.

All electrochemical measurements were performed on an Autolab electrochemical workstation (PGSTAT302N, Metrohm). The concentrations of the two electroactive probe (K_3_Fe(CN)_6_ and Ru(NH_3_)_6_Cl_3_) were 0.5 mM, and the supporting electrolyte solution was 0.05 M KHP. All of the electrochemical measurements were performed at room temperature. The scan rate for cyclic voltammetry (CV) tests was 100 mV/s. The parameters of differential pulse voltammetry (DPV) were as follows: step potential, pulse amplitude, pulse time, and interval time were 0.005 V, 0.05 V, 0.05 s, and 0.2 s, respectively. The error bars in the test were calculated as the standard deviations of the three measurements.

### 3.3. Preparation of VMSF/ErGO/SPCE

The SPCE was firstly electrochemically polished with continuous CV scanning from 0.4 V to 1.0 V for 10 cycles in 0.05 M H_2_SO_4_ solution. Afterwards, the electrode was thoroughly rinsed with ultrapure water and dried with N_2_ stream. Subsequently, 20 µL 0.1 mg/mL GO dispersion prepared by the conventional Hummers method was dropped on the surface of the working electrode and dried at 60 °C, termed as GO/SPCE.

The electrochemical-assisted self-assembly (EASA) method [54,55] was applied to grow VMSF on the GO/SPCE surface, and the preparation of the precursor solution is as follows: briefly, a mixture consisting of ethanol (20 mL), 0.1 M NaNO_3_ solution (20 mL, pH = 2.6), TEOS (3.05 mL), and CTAB (1.585 g), was firstly prepared; after being pre-hydrolyzed by stirring for 2.5 h at room temperature, the silica-based precursor was obtained.

After SPCE was immersed into the above precursor solution, a constant current density (−2.79 mA cm^−2^) was applied to the GO/SPCE for 10 s. The resulting electrode was immediately taken out, quickly rinsed with ultrapure water, and blow-dried with N_2_ stream, and aged at 80 °C for 10 h. Then, the electrode with surfactant micelle (SM) was obtained, termed as SM@VMSF/ErGO/SPCE. The VMSF/ErGO/SPCE electrode with open nanochannels was achieved after the removal of CTAB micelles from silica nanochannels by using 0.1 M HCl-ethanol solution by stirring for 5 min.

### 3.4. Electrochemical Detection of DOX

For DOX detection, phosphate buffer solution (PBS) (0.1 M, pH = 6) was used as the supporting electrolyte. DOX (*m* = 5.7998 g, MW = 579.98) was dissolved in 1 mL PBS solution, and 10 mM DOX mother liquor was prepared for gradient dilution in subsequent experiments. The electrochemical responses of DOX at different concentrations were recorded using CV or DPV. For real sample analysis, the serum and urine samples were diluted by a factor of 50 with the buffer without other complex pretreatments. The recovery tests of DOX in diluted human serum and urine were carried out using the standard addition method by comparing the determined concentration with the known spiked concentration.

## 4. Conclusions

In summary, we report on the fabrication of a disposable and portable electrochemical sensor by integrating VMSF and ErGO on a SPCE. Due to the electrostatic preconcentration and anti-fouling capacities of VMSF, excellent electrochemical activity of ErGO, and the disposable property of SPCE, highly sensitive and accurate determination of DOX in human serum and urine samples can be accomplished using the fabricated VMSF/ErGO/SPCE sensor. The proposed fabrication strategy of the sensor is simple, rapid, and controllable. The advantages of our VMSF/ErGO/SPCE sensor for DOX detection in human serum and urine samples include rapidity, portability, no complex sample pretreatment, and low sample consumption. We believe the proposed VMSF/ErGO/SPCE sensor can be applied for direct on-site analysis of more real samples by combination with intelligent instruments (e.g., smartphones).

## Figures and Tables

**Table 1:** Comparison of the analytical performances of different modified electrodes for the determination of DOX.

Electrode	Method	Linear Range (μM)	Sensitivity (μA μM^−1^)	LOD (nM)	Ref.
CDs/CeO_2_/SPCE	CV	0.2–20	1.39	90	[47]
AgNPs-CS-GCE	SWV	0.103–8.6	0.861	103	[48]
MB@MWCNTs/UiO-66-NH_2_/GCE	CV	0.1–75	0.0183	51	[49]
AuNRDs/1T-MoS_2_/SPE	DPV	0.01–9.5	0.895	2.5	[50]
SNPs@MOF/BNSs-Fc/GCE	SWV	0.01–10	0.641	2	[51]
BPPDNi/Pt:CO-NPs/CPE	SWV	0.5–300	0.0677	100	[52]
N-CNOs/GCE	DPV	2 × 10^−4^–10	2.49	0.06	[53]
VMSF/ErGO/GCE	DPV	0.001–20	7.815	0.77	[11]
VMSF/ErGO/SPCE	DPV	0.002–1 1–15	12.6 1.15	1	This work

CDs: carbon dots; CeO_2_: cerium oxide; AgNPs: silver nanoparticles; CS: chitosan; GCE: glassy carbon electrode; SWV: square wave voltammetry; MB: methylene blue; MWCNT: multi-walled carbon nanotubes; AuNRDs: gold nanorods; 1T-MoS_2_: Molybdenum disulfide; SPE: screen-printed electrode; SNPs: sulfur nanoparticles; MOF: metal–organic framework; BNSs: boron nanosheets; Fc: ferrocene; Pt:CO-NPs: Pt:Co nanoparticle; BPPDNi: bis(1,10-phenanthroline)(1,10-phenanthroline-5,6-dione)nickel(II) hexafluorophosphate; CPE: carbon-printed electrode; N-CNOs: nitrogen-doped carbon nano-onions.

**Table 2:** Determination of DOX in diluted human serum and urine ^a^.

Sample	Added (μM)	Found (μM)	RSD (%)	Recovery (%)
Human serum	0.0100	0.0103	3.1	103
	0.100	0.102	1.8	102
	1.00	0.999	1.7	99.9
	5.00	4.96	1.0	99.2
Urine	0.0100	0.0101	3.0	101
	0.100	0.104	2.8	104
	1.00	0.998	2.7	99.8
	5.00	5.21	1.6	104

^a^ Human serum and urine were diluted by 50 times with 0.1 M PBS (pH = 6).

## Data Availability

The data presented in this study are available on request from the corresponding author.

## Supplementary Materials

The following supporting information can be downloaded at: https://www.mdpi.com/article/10.3390/molecules27248640/s1, Figure S1: Effect of scan rate on the CV responses; Figure S2: Effect of growth time of VMSF on the detection performance; Figure S3: Optimization of preconcentration time and pH value of supporting electrolyte.
