# Supplementary material for: Anti-Biofouling Electrochemical Sensor Based on the Binary Nanocomposite of Silica Nanochannel Array and Graphene for Doxorubicin Detection in Human Serum and Urine Samples
Source: Molecules. 2022 Dec 7;27(24):8640. doi: 10.3390/molecules27248640 (PMC9786716; doi:10.3390/molecules27248640)
Supplement: Supplementary file 1 [file molecules-27-08640-s001.zip › molecules-2041960-supplementary.pdf]

Supporting Information to

Anti-Biofouling Electrochemical Sensor Based on the Binary Nanocomposite of Silica Nanochannel Array and Graphene for Doxorubicin Detection in Human Serum and Urine Samples

Ning Lv [1,†], Xun Qiu [2,†], Qianqian Han [3], Fengna Xi [3], Yina Wang [4,*] and Jun Chen [2,*]

[1] Department of Pharmacy, The First Affiliated Hospital, College of Medicine, Zhejiang University, 79 Qingchun Road, Hangzhou 310003, China; lvn1987@zju.edu.cn
[2] Key Laboratory of Integrated Oncology and Intelligent Medicine of Zhejiang Province, Department of Hepatobiliary and Pancreatic Surgery, Affiliated Hangzhou First People's Hospital, Zhejiang University School of Medicine, Hangzhou 310006, China; 3180103169@zju.edu.cn
[3] Department of Chemistry, Zhejiang Sci-Tech University, Hangzhou 310018, China; hanqq1007@126.com (Q.H.); fengnaxi@zstu.edu.cn (F.X.)
[4] Department of Medical Oncology, The First Affiliated Hospital, College of Medicine, Zhejiang University, 79 Qingchun Road, Hangzhou 310003, China
* Correspondence: 1503099@zju.edu.cn (Y.W.); herewego008@163.com (J.C.)
† These authors contributed equally to this work.

Table of Contents

S1. Effect of scan rate on the CV responses

S2. Effect of growth time of VMSF on the detection performance

S3. Optimization of preconcentration time and pH value of supporting electrolyte

S1. Effect of scan rate on the CV responses

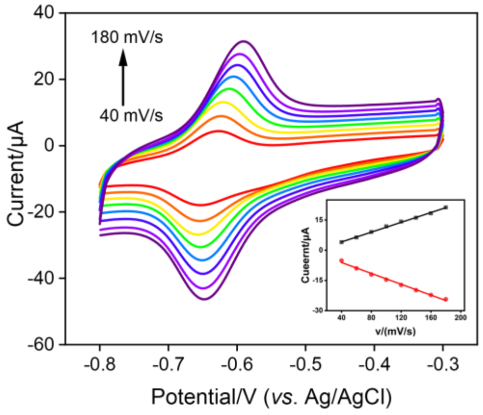

Figure S1 Effect of the scan rate on the CV responses obtained from the VMSF/ErGO/SPCE in 0.1 M PBS (pH = 6.0) containing 10 μM DOX. The inset is the dependence of anodic and cathodic peak currents on scan rate.

S2. Effect of growth time of VMSF on the detection performance

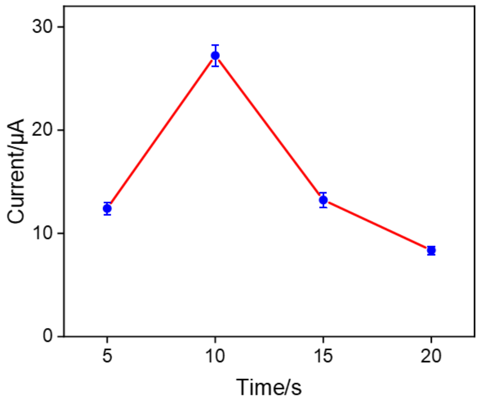

Figure S2 Effect of growth time of VMSF on the current response of 10 μM DOX in 0.1 M PBS (pH = 6.0). The error bars represent the standard deviations of three measurements.

S3 Optimization of preconcentration time and pH value of supporting electrolyte

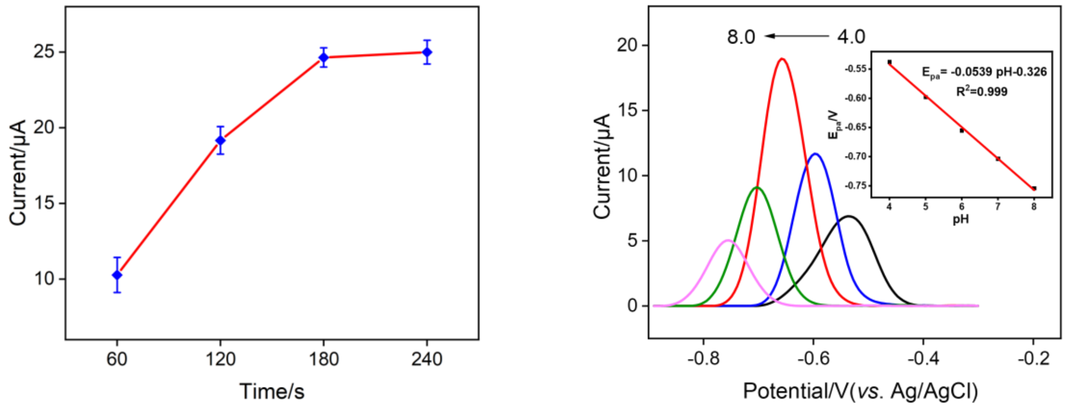

(**a**) (**b**)

Figure S3 (**a**) Effect of stirring time on the current response of 10 μM DOX in 0.1 M PBS (pH = 6.0). (**b**) DPV curves of VMSF/ErGO/SPCE in 0.1 M PBS solution containing 10 μM DOX at different pH values. The inset shows the linear dependence of anodic peak potential (E_{pa}) on pH value. The error bars represent the standard deviations of three measurements.
